# Statistical Report of One Hundred and Seven Cases of Labour, Occurring in the Lying-in Department of the Philadelphia Hospital (Blockley)

**Published:** 1844-02-24

**Authors:** George H. Beaumont

**Affiliations:** Late Resident Physician


### THE MEDICAL EXAMINER,

### And Record of Medical Science.

Vol. VII.] PHILADELPHIA, SATURDAY, FEBRUARY 24, 1844. [No. 4

### STATISTICAL REPORT OF ONE HUNDRED AND SEVEN CASES OF LABOUR, OCCURRING IN THE LYING-IN DEPARTMENT OF THE PHILADELPHIA HOSPITAL (BLOCKLEY.)

By George H. Beaumont, M. D.,

*Late Resident Physician.*

| *NAME.* | *Colour.* | *Age.* | *Presentation of head.* | *Presentation of breech.* | *Hemorrh. before deliv.* | *Hemorrh. after deliv.* | *Retained placenta.* | *Convulsions.* | *Forceps.* | *Craniotomy.* | *Died.* | *Recovered.* | *REMARKS.* |
|---|---|---|---|---|---|---|---|---|---|---|---|---|---|
| Jane Wittington, - - - | B. | 24 | 2 v. | 0 | 0 | 0 | 0 | 0 | 0 | 0 | 0 | 1 | |
| Sarah Warmingham, - - | W. | 26 | 1 " | 0 | 0 | 0 | 0 | 0 | 0 | 0 | 0 | 1 | |
| Elizabeth Pancost, - - | B. | 19 | 1 " | 0 | 0 | 0 | 0 | 0 | 0 | 0 | 0 | 1 | |
| Teresa Austin, - - - | W. | 24 | 1 " | 0 | 0 | 0 | 0 | 0 | 0 | 0 | 0 | 1 | |
| Hannah Yocum, - - - | W. | 20 | 1 " | 0 | 0 | 0 | 0 | 0 | 0 | 0 | 0 | 1 | |
| Julia Allen, - - - | B. | 24 | 1 " | 0 | 0 | 0 | 0 | 0 | 0 | 0 | 0 | 1 | |
| Bridget Smith, - - - | W. | 30 | 1 " | 0 | 0 | 0 | 0 | 0 | 0 | 0 | 0 | 1 | |
| Margaret Price, - - - | W. | 30 | 1 " | 0 | 0 | 0 | 0 | 0 | 0 | 0 | 0 | 1 | |
| Mary Hannan, - - - | W. | 26 | 1 " | 0 | 0 | 0 | 0 | 0 | 0 | 0 | 0 | 1 | |
| Julia Ann Hoffman, - - | W. | 29 | 1 " | 0 | 0 | 0 | 0 | 0 | 0 | 0 | 0 | 1 | |
| Catharine Wagner, - - | W. | 29 | 2 " | 0 | 0 | 0 | 0 | 0 | 0 | 0 | 0 | 1 | |
| Frances Moor, - - - | B. | 21 | 1 " | 0 | 0 | 0 | 0 | 0 | 0 | 0 | 0 | 1 | |
| Ann Elizabeth Fricke, - - | W. | 19 | 1 " | 0 | 0 | 0 | 0 | 0 | 0 | 0 | 0 | 1 | |
| Sarah Gilbert, - - - | W. | 25 | 1 " | 0 | 0 | 0 | 0 | 0 | 0 | 0 | 0 | 1 | |
| Rachael Moor, - - - | B. | 18 | 1 " | 0 | 0 | 0 | 0 | 0 | 0 | 0 | 0 | 1 | |
| Mary Jane Janvier, - - | W. | 20 | 1 " | 0 | 0 | 0 | 0 | 0 | 0 | 0 | 0 | 1 | |
| Maria Forte, - - - | W. | 22 | 1 " | 0 | 0 | 0 | 0 | 1 | 0 | 0 | 0 | 1 | |
| Mary Whales, - - - | W. | 22 | 1 " | 0 | 0 | 0 | 0 | 0 | 0 | 0 | 0 | 1 | |
| Catharine McArty, - - | W. | 27 | 1 " | 0 | 0 | 0 | 0 | 0 | 0 | 0 | 0 | 1 | |
| Louisa Fricke, - - - | W. | 22 | 1 " | 0 | 0 | 0 | 0 | 0 | 0 | 0 | 0 | 1 | |
| Mary Smith, - - - | B. | 22 | 1 " | 0 | 0 | 0 | 0 | 0 | 0 | 0 | 0 | 1 | |
| Susannah Martin, - - - | W. | 28 | 1 " | 0 | 0 | 0 | 0 | 0 | 0 | 0 | 0 | 1 | |
| Sarah Brett, - - - | W. | 33 | 1 " | 0 | 0 | 0 | 0 | 0 | 0 | 0 | 0 | 1 | |
| Elizabeth Bagwell, - - | B. | 25 | 1 " | 0 | 0 | 0 | 0 | 0 | 0 | 0 | 0 | 1 | |
| Abigail Edwards, - - - | W. | 15 | 1 " | 0 | 0 | 0 | 0 | 0 | 0 | 0 | 0 | 1 | |
| Phoebe Reeves, - - - | W. | 32 | 1 " | 0 | 0 | 0 | 0 | 0 | 0 | 0 | 0 | 1 | |
| Elizabeth Ball, - - - | W. | 20 | 1 " | 0 | 0 | 0 | 0 | 0 | 0 | 0 | 0 | 1 | |
| Ann Sweeny, - - - | W. | 25 | 1 " | 0 | 0 | 0 | 0 | 0 | 0 | 0 | 0 | 1 | |
| Emma Smith, - - - | B. | 18 | 1 " | 0 | 0 | 0 | 0 | 0 | 0 | 0 | 0 | 1 | |
| Ann Jones, - - - | B. | 23 | 1 " | 0 | 0 | 0 | 0 | 0 | 0 | 0 | 0 | 1 | |
| Nancy Myers, - - - | W. | 31 | 1 " | 0 | 0 | 0 | 0 | 0 | 0 | 0 | 0 | 1 | |
| Susan Brannan, - - - | W. | 23 | 1 " | 0 | 0 | 0 | 0 | 0 | 0 | 0 | 0 | 1 | |
| Hannah McGui, - - - | B. | 38 | 1 " | 0 | 0 | 0 | 0 | 0 | 0 | 0 | 0 | 1 | |
| Ellen Young, - - - | W. | 30 | 1 " | 0 | 0 | 0 | 0 | 0 | 0 | 0 | 0 | 1 | |
| Emma Garwood, - - . | W. | 22 | 1 " | 0 | 0 | 0 | 0 | 0 | 0 | 0 | 0 | 1 | |
| Ann Essler, - - - | W. | 25 | 1 " | 0 | 0 | 0 | 0 | 0 | 0 | 0 | 0 | 1 | |
| Nancy Underwood, - - | B. | 22 | 3 " | 0 | 0 | 0 | 0 | 0 | 0 | 0 | 0 | 1 | |
| Elizabeth Smith, - - - | B. | 23 | 1 " | 0 | 0 | 0 | 0 | 0 | 0 | 0 | 0 | 1 | |
| Emma Cook, - - - | B. | 25 | 1 " | 0 | 0 | 0 | 0 | 0 | 0 | 0 | 0 | 1 | |
| Hannah Porter, - - - | W. | 18 | 1 " | 1 | 0 | 0 | 0 | 0 | 0 | 0 | 0 | 1 | |
| Ann Kane, - - - | W. | 35 | 1 " | 0 | 0 | 0 | 0 | 0 | 0 | 0 | 0 | 1 | |
| Catharine Brown, - - | W. | 25 | 1 " | 0 | 0 | 0 | 0 | 0 | 0 | 0 | 0 | 1 | |
| Mary Hughs, - - - | W. | 19 | 1 " | 0 | 0 | 0 | 0 | 0 | 0 | 0 | 0 | 1 | |
| Margaret Ludlow, - - | W. | 22 | 1 " | 0 | 0 | 0 | 0 | 0 | 0 | 0 | 0 | 1 | |
| Mary Wilson, - - - | W. | 36 | 1 " | 0 | 0 | 0 | 0 | 0 | 0 | 0 | 0 | 1 | |
| Mary McArty, - - - | W. | 21 | 1 " | 0 | 0 | 0 | 0 | 0 | 0 | 0 | 0 | 1 | |
| Matilda Davis, - - - | W. | 18 | 1 " | 0 | 0 | 0 | 0 | 0 | 0 | 0 | 0 | 1 | |

| NAME. | Colour. | Age. | Presentation of head. | Presentation of breech. | Hemorrh. before deliv. | Hemorrh. after deliv. | Retained placenta. | Convulsions. | Forceps. | Craniotomy. | Died. | Recovered. | REMARKS. |
|---|---|---|---|---|---|---|---|---|---|---|---|---|---|
| Hannah Groves, - | W. | 28 | 2 vert. | 0 | 0 | 0 | 0 | 0 | 0 | 0 | 0 | 1 | |
| Margaret Gray, - | W. | 20 | 1 " | 0 | 0 | 0 | 0 | 0 | 0 | 0 | 0 | 1 | |
| Mary Foley, - | W. | 24 | 1 " | 0 | 0 | 0 | 0 | 0 | 0 | 0 | 0 | 1 | |
| Hannah Friel, - | W. | 23 | 1 " | 0 | 0 | 0 | 0 | 0 | 0 | 0 | 0 | 1 | |
| Margaret McNulty, | W. | 31 | 1 " | 0 | 0 | 0 | 0 | 0 | 1 | 0 | 0 | 1 | |
| Mary Jane Holts, - | W. | 18 | 1 " | 0 | 0 | 0 | 0 | 0 | 0 | 0 | 0 | 1 | |
| Mary Kale, - | W. | 33 | 2 " | 0 | 0 | 0 | 0 | 0 | 0 | 0 | 1 | 0 | 4 days after of puerp. peritonitis |
| Mary Winterbottom, | W. | 38 | 1 " | 0 | 0 | 0 | 0 | 0 | 0 | 0 | 0 | 1 | |
| Alice Mumford, - | W. | 30 | 1 " | 0 | 0 | 0 | 0 | 0 | 0 | 0 | 1 | 0 | 11 days after of typhus. |
| Catharine Hill, - | W. | 36 | 1 " | 0 | 0 | 0 | 0 | 0 | 0 | 0 | 0 | 1 | Twins. |
| Barbara Bryant, - | W. | 18 | 3 " | 0 | 0 | 0 | 0 | 0 | 0 | 0 | 0 | 1 | |
| Hannah Bumm, - | W. | 20 | 1 " | 0 | 0 | 0 | 0 | 0 | 0 | 0 | 1 | 0 | 7 days after of puerp. peritonitis |
| Elizabeth Parker, - | W. | 24 | 1 " | 0 | 0 | 0 | 0 | 0 | 0 | 0 | 0 | 1 | |
| Charlotte Higs, - | W. | 28 | 1 " | 0 | 0 | 0 | 0 | 0 | 0 | 0 | 1 | 0 | 5 days after of puerp. peritonitis |
| Ellen Johnson, - | W. | 23 | 1 " | 0 | 0 | 0 | 0 | 0 | 0 | 0 | 0 | 1 | |
| Matilda Kane, - | W. | 24 | 1 " | 0 | 0 | 0 | 0 | 0 | 0 | 0 | 0 | 1 | |
| Mary Ann Nixon, | W. | 28 | 1 " | 0 | 0 | 0 | 0 | 0 | 0 | 0 | 0 | 1 | |
| Catharine A. Scott, | W. | 21 | 1 " | 0 | 0 | 0 | 0 | 0 | 0 | 0 | 0 | 1 | |
| Catharine Young, - | W. | 36 | 1 " | 0 | 0 | 0 | 0 | 0 | 0 | 0 | 0 | 1 | |
| Sarah Hildebrand, | W. | 20 | 1 " | 0 | 0 | 0 | 0 | 0 | 0 | 0 | 1 | 0 | 6 days after of puerp. peritonitis |
| Susan Williams, - | W. | 26 | 1 " | 0 | 0 | 0 | 0 | 0 | 0 | 0 | 0 | 1 | |
| Mary Donnaldson, | W. | 21 | 1 " | 0 | 0 | 0 | 0 | 0 | 0 | 0 | 1 | 0 | 6 days after of puerp. peritonitis |
| Mary Ann Thompson, | W. | 28 | 1 " | 0 | 0 | 0 | 0 | 0 | 0 | 0 | 0 | 1 | |
| Ann Russel, - | W. | 22 | 1 " | 0 | 0 | 0 | 0 | 0 | 0 | 0 | 0 | 1 | |
| Mary McArty, - | W. | 00 | 1 " | 0 | 0 | 0 | 0 | 0 | 0 | 0 | 1 | 0 | 5 days after of puerp. peritonitis |
| Elizabeth Martin, - | W. | 25 | 1 " | 0 | 0 | 0 | 0 | 0 | 0 | 0 | 0 | 1 | |
| Sarah Dockerty, - | W. | 20 | 2 " | 0 | 0 | 0 | 0 | 0 | 0 | 0 | 0 | 1 | |
| Ellen Mina Hall, - | W. | 00 | 1 " | 0 | 0 | 0 | 0 | 0 | 0 | 0 | 0 | 1 | |
| Martha Jane Parker, | W. | 23 | 1 " | 0 | 0 | 0 | 0 | 0 | 0 | 0 | 0 | 1 | |
| Elizabeth Scott, - | W. | 20 | 2 " | 0 | 0 | 0 | 0 | 0 | 0 | 0 | 0 | 1 | |
| Ann Stewart, - | W. | 30 | 1 " | 0 | 0 | 0 | 0 | 0 | 0 | 0 | 0 | 1 | |
| Mary Pride, - | W. | 19 | 0 " | 4 | 0 | 0 | 0 | 0 | 0 | 0 | 0 | 1 | |
| Matilda Knox, - | W. | 22 | 1 " | 0 | 0 | 0 | 0 | 0 | 0 | 0 | 0 | 1 | |
| Matilda Robinson, | W. | 26 | 1 " | 0 | 1 | 0 | 0 | 0 | 0 | 0 | 0 | 1 | |
| Louisa Scott, - | W. | 28 | 1 " | 0 | 1 | 0 | 0 | 0 | 0 | 0 | 0 | 1 | |
| Elizabeth McAffee, | W. | 24 | 4 " | 0 | 0 | 0 | 0 | 0 | 0 | 0 | 0 | 1 | |
| Elizabeth Burge, - | W. | 18 | 1 " | 0 | 0 | 0 | 0 | 0 | 0 | 0 | 0 | 1 | |
| Elizabeth Crowley, | W. | 30 | 1 " | 0 | 0 | 0 | 0 | 0 | 0 | 0 | 0 | 1 | |
| Lucy Pingston, - | B. | 21 | 1 " | 0 | 0 | 0 | 0 | 0 | 0 | 0 | 0 | 1 | |
| C. Randolph Wilson, | B. | 22 | 1 " | 0 | 0 | 0 | 0 | 0 | 0 | 0 | 0 | 1 | |
| Beulah Dietz, - | W. | 22 | 1 " | 0 | 0 | 0 | 0 | 0 | 0 | 0 | 0 | 1 | |
| Ann Tierney, - | W. | 23 | 1 " | 0 | 0 | 0 | 0 | 0 | 0 | 0 | 0 | 1 | |
| Mary Wright, - | W. | 20 | 1 " | 0 | 0 | 0 | 0 | 0 | 0 | 0 | 0 | 1 | |
| Elizabeth Griffith, | W. | 23 | 1 " | 0 | 0 | 0 | 0 | 0 | 0 | 0 | 0 | 1 | |
| Ann Deverney, - | W. | 25 | 2 " | 0 | 0 | 0 | 0 | 0 | 0 | 0 | 0 | 1 | |
| Sarah Peters, - | B. | 33 | 1 " | 0 | 0 | 0 | 0 | 0 | 0 | 0 | 0 | 1 | |
| Susan Koons, - | W. | 24 | 1 " | 0 | 0 | 0 | 0 | 0 | 0 | 0 | 0 | 1 | |
| Sarah Sharpe, - | W. | 22 | 1 " | 0 | 0 | 0 | 1 | 0 | 0 | 0 | 0 | 1 | |
| Caroline Clarke, - | W. | 20 | 1 " | 0 | 0 | 0 | 0 | 0 | 0 | 0 | 0 | 1 | |
| Maria Jones, - | B. | 20 | 1 " | 0 | 0 | 0 | 0 | 1 | 0 | 0 | 0 | 1 | |
| Margaret Richardson, | W. | 18 | 1 " | 0 | 0 | 0 | 0 | 0 | 0 | 0 | 0 | 1 | |
| Sarah ———, - | W. | 22 | 1 " | 0 | 0 | 0 | 0 | 0 | 0 | 0 | 0 | 1 | |
| Jane Wood, - | B. | 24 | 1 " | 0 | 0 | 0 | 0 | 0 | 0 | 0 | 0 | 1 | |
| Ellen Crawson, - | W. | 22 | 4 of fa. | 0 | 0 | 0 | 0 | 0 | 0 | 0 | 0 | 1 | |
| Emma Jackson, - | W. | 22 | 2 vert. | 0 | 0 | 0 | 0 | 0 | 1 | 0 | 0 | 1 | |
| Elizabeth Jones, - | W. | 17 | 1 " | 0 | 0 | 0 | 0 | 0 | 0 | 0 | 0 | 1 | |
| Elizabeth Moore, - | W. | 18 | 0 " | 1 of ft. | 0 | 0 | 0 | 0 | { Vectis used. | 0 | 0 | 1 | |
| Margaret Watson, | W. | 24 | 1 " | 2 of br. | 0 | 0 | 0 | 0 | 0 | 0 | 0 | 1 | |
| Margaret Richardson, | W. | 21 | 1 " | " | 0 | 0 | 0 | 0 | 0 | 0 | 0 | 1 | |
| Julia Ferguson, - | W. | 29 | 1 " | " | 0 | 0 | 0 | 0 | 0 | 0 | 0 | 1 | |
| | 107 | | 103 | 4 | 2 | 1 | 1 | 2 | 2 | 0 | 7 | 100 | |

